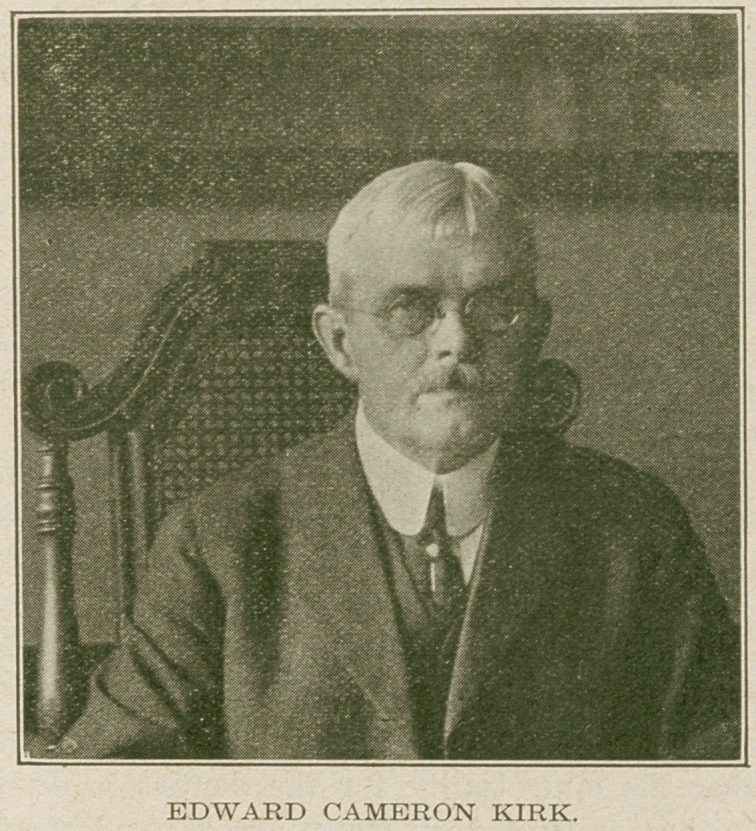# The New Building of Thomas W. Evans Museum and Dental Institute School of Dentistry, University of Pennsylvania

**Published:** 1915-03

**Authors:** 


### THE NEW BUILDING OF THOMAS W. EVANS MUSEUM AND DENTAL INSTITUTE SCHOOL OF DENTISTRY, UNIVERSITY OF PENNSYLVANIA.

The dedication of the Evans Dental Institute School of Dentistry of the University of Pennsylvania on Monday, February 22nd, marks an epoch in the history of dental education. It not only formally opens the largest and best equipped plant in the world, devoted exclusively to the teaching of Dental Science, but above all it demonstrates the wisdom of modern educators in their endeavor to strengthen the worthy schools already established rather than to found new ones, which so often have a hard struggle for existence, and which, because of the lack of necessary financial support do inferior work. The standard which the Evans Institute will be able to maintain will be of the very highest type, and should result in carrying out, in the most effective manner possible, the wishes of the great Philadelphia philanthropist, by whose name the Institute and School will hereafter be known.

The new building is in the Tudor style of architecture which prevailed in the time of Henry VIII and might be described as Collegiate Gothic. It is constructed of Indiana limestone and hard-burnt brick. The building has a frontage on Spruce Street of 242 feet, and a depth to Irving Street along Fortieth Street of 161 feet. It is built in the form of the letter H and has three stories over a high basement. The benefaction of Dr. Evans includes this building with its equipment and a substantial endowment fund.

Among the commanding features of the building is the square tower, through which is the main entrance, in the center of the Spruce Street front. It is thirty-eight feet square, rising to eighty-four feet. In the center of the tower, beginning at the second story and reaching almost to the top of the third floor, is a large window, which lights the library on the second floor.

The Evans Museum occupies the east half of the Spruce Street front, and is as nearly fire and burglar proof as modern science can make it. This houses the priceless Evans collection.

In the west end of the Spruce Street front are the offices of the Dean of the Institution, and the Board room. The rest of the ground floor is divided into class-rooms, laboratories, and prosthetic clinic rooms, the entire north wing being devoted to this purpose. To the right and left of the central hallway, which extends from the first floor to the roof, are rooms for various phases of clinical dental service, radiography, photography, instructors' rooms, etc., and a model dental office.

Another of the imposing features of the building is the large operative clinic hall in the north wing on the second floor. This occupies practically the entire wing on Irving Street and is two hundred feet long by forty-eight feet wide.

This clinic room is thirty feet high, with a glass wall on the north side; the roof for a distance of about ten feet is also glass, giving all the daylight possible. The floor is covered with battleship linoleum. A gallery on the south side contains students' lockers. The room is furnished with 135 chairs; each chair is equipped with electric service for power and light, gas, compressed air and water service to each chair. In the south wing on the same floor is located the library, which extends up through the third floor of the tower with galleries on each side, and to the east the main

BASEMENT FLOOR PLAN

lecture hall, eighty-seven by forty-three feet, to the west three small laboratories and a lecture room for post graduate work, in Porcelain and Gold Inlay and Crown and Bridge work. One of the principal objects of the Institute will be the encouragement of research work, and a number of rooms for that purpose are on the second and third floors. Pharmacology, Histology and Bacteriology are all provided for.

The main stairway ends at the second floor, in a large hall open to the roof. The side walls, of this hallway are in pinkish gray stone, and the ceiling is of metal and plaster, formed and painted to represent the carved wooden ceilings of the Tudor period.

Two large laboratories, excellently lighted, occupy the third floor of the south wing. Other rooms for research work and post-graduate instruction are in the western end.

In the basement are locker rooms for the students, laboratories for prosthetic technics, metallurgy and lecture rooms for first year men, also a restaurant for students and faculty.

The power house adjoins the building on the north. This contains two boilers with a capacity of 400 horse power. The engines and electric generators are capable of producing 240 kilowatts, and will furnish the lighting, heating and ventilating for the laboratories, clinics and other rooms in the building.

The Dental School was organized in 1878 and is the most cosmopolitan of the departments of the University, its students usually representing about twenty-five foreign countries and almost every state of the Union. It now has a teacher staff of eighty-three professors and instructors, and six hundred and sixty-five students. The school operates a free dispensary, in which about 40,000 cases are treated annually.

When the school was first organized, it occupied for a short time a room in the old Medical Hall (now Logan Hall), and subsequently quarters in the Hare Laboratory

FIRST FLOOR PLAN

of Chemistry at Thirty-sixth and Spruce Streets, but in 1896 it removed to a building especially constructed for it. There its growth has been remarkable, and it has long since outgrown these quarters. It now occupies its fourth home.

By concurrent action of the Trustees of The Thomas W. Evans Museum and Institute Society and the University of Pennsylvania an agreement between them was executed on Saturday, June 15, 1912, by the provisions of which a co-operative affiliation of the two institutions was consummated, so that the resources of both have been utilized in the creation of a dental school to be carried on "as such institutions of learning are now conducted in Philadelphia, and not inferior to any already established," as provided in the will of the late Dr. Thomas W. Evans, an eminent scientist and dentist who practised in Europe, but who was born in Philadelphia, and lived in a house which stood where the new building bearing his name now stands.

The dedicatory exercises were attended by distinguished alumni and dentists from all parts of this country and abroad. The day was beautiful and the guests, officers, alumni and students gathered in the old Dental Hall and formed a procession which marched to the new building where the dedication exercises were held in the beautiful clinic room. Addresses were made by Dr. Wm. H. Mummery of London, England; Dr. Wm. Simon, of Baltimore and Dr. E. C. Kirk, of Philadelphia. Honorary Degrees were then conferred on the following men by the University Provost: On Mr. John T. Windrim, the degree of Master of Science in Architecture, in appreciation of his work as architect of the building; on Dr. E. C. Kirk, the degree of Doctor of Laws, in recognition of his services as designer of the building, teacher, and scientist; on Dr. E. T. Darby, the degree of Doctor of Laws, for his distinguished career as a dental teacher; on Dr. Truman W. Brophy, the degree of Doctor of Science, for his distinguished services as an oral surgeon and teacher; on Dr. William Simon, the degree of Doctor of Science for his long career as a teacher

SECOND FLOOR PLAN

THIRD FLOOR PLAN

and writer on chemical science; on Dr. Edward H. Angle, the degree of Doctor of Science, for the eminent services rendered by him in the development of the art and science of orthodontia; on John Howard Mummery, of London, England, the degree of Doctor of Science for his great work in dental science; on Dr. E. C. Godon, of Paris, France, the degree of Doctor of Science, for his distinguished work as an author, educator and professional organizer; on Dr. Eugene S. Talbot, the degree of Doctor of Science, for his distinguished work as author and contributor to dental literature; on Dr. Wilhelm Dieck, of Berlin, Germany, the degree of Doctor of Science, for his services as author, teacher and dental organizer; on Dr. G. V. Black, the degree of Doctor of Science, for his long and eminent services as teacher, writer and dental scientist.

The second day of the dedication exercises were given over to clinics, inspection of the building, and a most enthusiastic alumni meeting in the old Dental Hall, of which they took formal leave, in a banquet and a number of excellent speeches.

Thomas W. Evans, who gave the money for the erection of this great building, acquired his money while practicing dentistry in Paris, France. He was a native of Philadelphia. He was born in Philadelphia, December 23, 1823 and died in Paris, November 14, 1897. He got his first notion of dentistry from his occupation as a worker in a surgical instrument house, where he made gold alloys and plate materials for dentists. He studied dentistry in the office of Dr. J. D. White, in Philadelphia, and later graduated from Jefferson Medical College. In 1847 he went to Paris and associated himself with Dr. C. S. Brewster, an American dentist. In 1850 he left Dr. Brewster and opened an office for himself in Paris. By his personality and technical skill he soon established himself and became the most popular dentist of his time. His great capacity as a ''mixer'' with royalty and great people made him the most talked of American dentist. He was the friend and dentist of many

European monarchs and especially of the French nobility. His most notorious escapade was the carrying of the Empress out of Paris to Calais, when she escaped to England. The carriage in which this was accomplished is to be put on exhibition in the museum of the new building in Philadelphia. His fortune was amassed by real estate speculations during the rebuilding of Paris by Napoleon

THOMAS W. EVANS.

the Third. He did a great deal of public charity work. He was devoted to his profession and gave largely of his time and money to promote the profession in France. He believed in spreading abroad in every possible manner any professional matter that came to him from any and every source. It seems too bad that he could not have seen the beautiful building erected with his money. The building with its equipment has cost between seven and eight hundred thousand dollars and there will be several hundred thousand dollars left for endowment purposes. It is said that the estate was worth five or six million dollars before Dr. Evans died, but through bad management, the trustees only secured about a million and a half dollars. The will requires that a monument costing two hundred and fifty thousand dollars must be erected in memory of his life.

EDWARD CAMERON KIRK.

This monument money could be more usefully spent by using it to erect another good dental educational building somewhere, but it seems the trustees have been left no discretion in the matter of investing this money. The dental profession should honor Dr. Kirk for his acumen in saving from the lawyers and court expenses, as much of this estate as he has done. It is a great enterprise.

## Figures and Tables

**Figure f1:**
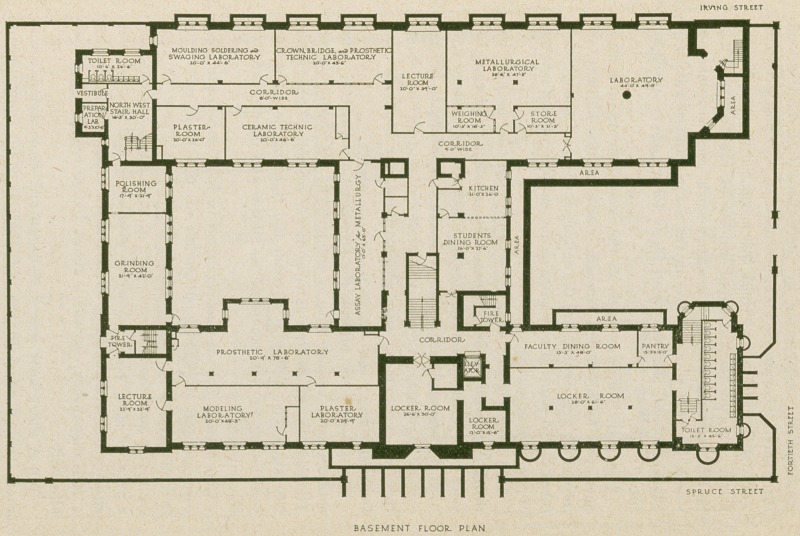


**Figure f2:**
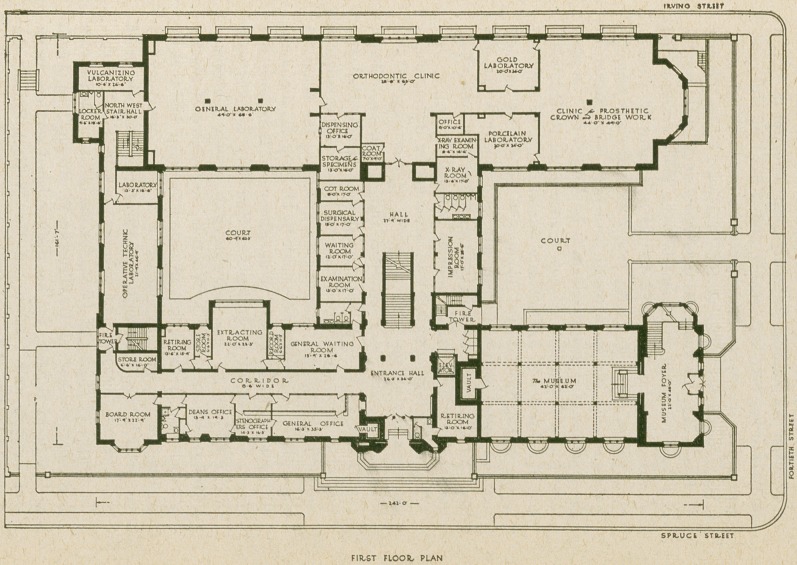


**Figure f3:**
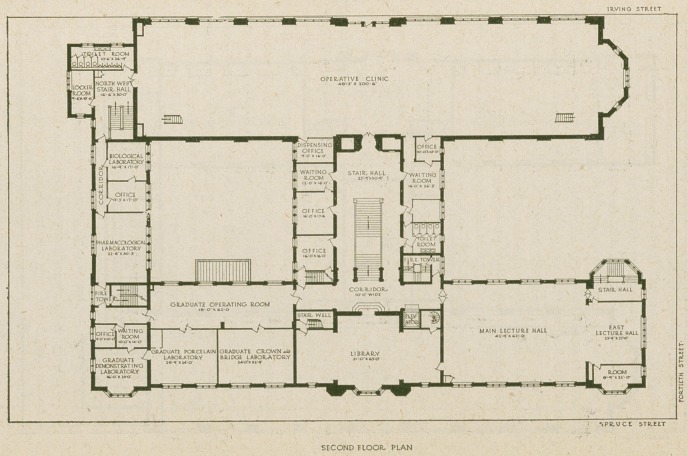


**Figure f4:**
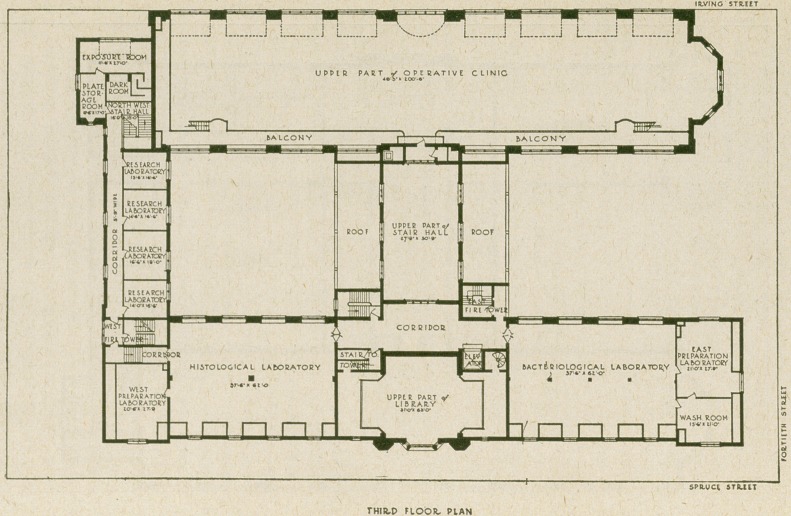


**Figure f5:**
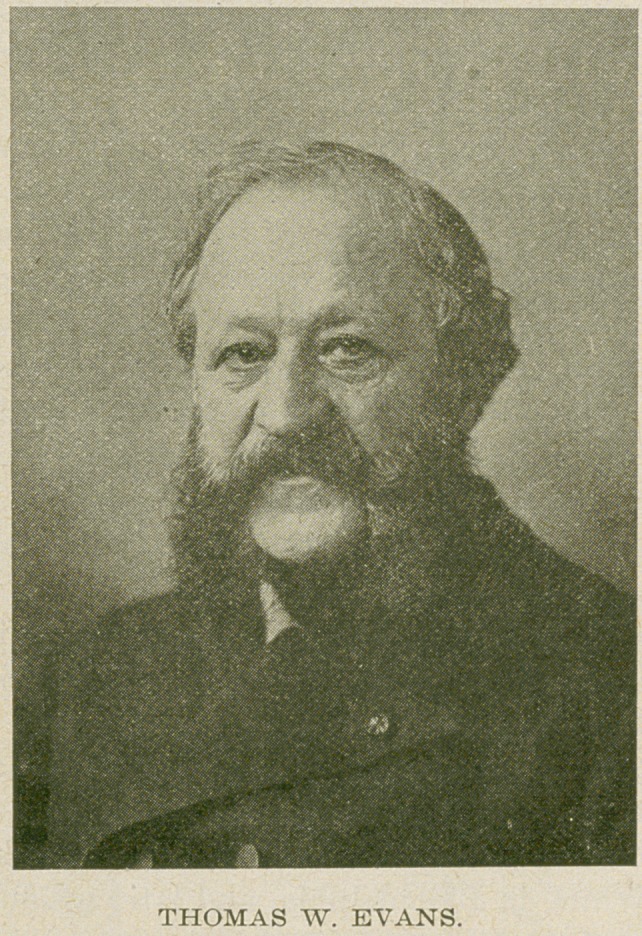


**Figure f6:**